# Regional right ventricular wall motion in tetralogy of fallot: a three dimensional analysis

**DOI:** 10.1186/1532-429X-11-S1-P118

**Published:** 2009-01-28

**Authors:** Michael Morcos, Christopher S Kirk, Florence Sheehan

**Affiliations:** University of Washington, Seattle, WA USA

**Keywords:** Wall Motion, Right Ventricle, Free Wall, Contraction Pattern, Right Ventricle Function

## Background

We previously reported an abnormal contraction pattern in the right ventricle (RV) in tetralogy of Fallot (TOF) patients by measuring the regional contribution to global stroke volume (rSV) at 20 cross sections. However the rSV method only measures regions spanning the RV from apex to base. Therefore we tested a second method that allows regions of interest to be defined for RV function.

## Purpose

We wanted to develop a method that enables detailed regional RV wall motion analysis that is capable of producing a wide variety of user-specific regional designation.

## Methods

We reconstructed the RV in 3D from manually traced borders in 20 repaired TOF patients and 9 normal (NL) subjects from magnetic resonance images. Wall motion was measured using the Centersurface (CS) method as the local orthogonal distance between the RV endocardium at end diastole and end systole in 10 regions on the RV free wall, the septum, and the outflow track. The free wall was divided into apical, middle, and basal levels; each level was further subdivided into inferior, middle, and superior regions. Regional function by both methods was compared between TOF and NL groups.

## Results

**Figure 1:**
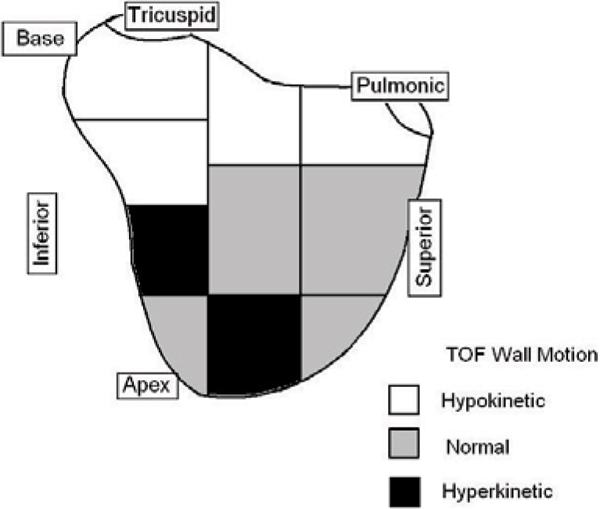
Figure 1

Both methods showed that TOF patients have reduced function at the base and increased function at the apex compared to NL (Figure 1). In both groups motion was lower in superior than in mid or inferior regions (p < 0.001 for all).

## Conclusion

The rSV and CS methods yielded similar results when comparing function in regions spanning the RV from apex to base. Only the CS method enabled comparison of function in the subdivided regions. CS provides a more comprehensive analysis of regional RV wall motion due to its flexibility in defining the region of interest.

